# Understanding Australian female chiropractors’ experiences of inappropriate patient sexual behaviour: a study using Interpretive Phenomenological Analysis

**DOI:** 10.1186/s12998-021-00394-1

**Published:** 2021-09-15

**Authors:** Stanley Innes, Laura Maurice, Michele Lastella, Catherine O’Mullan

**Affiliations:** 1 College of Science, Health, Engineering and Education (SHEE) , Murdoch University, Murdoch, Australia; 2 School of Health, Medical and Applied Sciences, CQUniversity Australia, Bundaberg Campus, Norman Gardens, Australia

**Keywords:** Workplace violence, Healthcare workers, Chiropractic, Female

## Abstract

**Introduction:**

Female practitioners are often subjected to inappropriate patient sexual behaviour (IPSB). Adverse consequences of such sexual harassment include for the practitioner psychological stress effects and negative work-related consequences that contributes to career dissatisfaction and burnout. Confronting the issue within the healthcare context has been shown to be problematic because practitioners feel an obligation to protect the therapeutic relationship above their own personal discomfort. There is an absence of research on this topic with respect to female chiropractors and we proposed a qualitative study aimed to explore female chiropractors lived experiences of managing incidents of IPSB.

**Method:**

An Interpretive Phenomenological Analysis methodology was chosen for this study. In June and July of 2018 female chiropractors in Western Australian were recruited via Facebook sites and invited to participate in face-to-face interviews for an Honours degree study exploring the lived experience of IPSB.

**Results:**

Participants were seven female chiropractors currently practicing in Western Australia, who had experienced an incident of IPSB. Four super-ordinate themes emerged from the analysis; (1) familiar but inarticulable, (2) the cost of conflict, (3) I’m used to it, and (4) the element of surprise. Overall, the participants recognised the incidents as inappropriate but chose to ignore the situation as a means to avoid conflict in the treatment room. Recommendations are made to better manage IPSB including greater patient awareness of appropriate behaviour, specific curriculum content and assertiveness training in undergraduate programs and continuing professional education, as well as the creation of ethical guidelines for patient behaviour by regulatory bodies.

**Conclusion:**

This is the first study to give a forum for female chiropractors to discuss their experiences of IPSB. The domain of private practice is not immune to incidents IPSB and although similar to day-to-day non-clinical life is nonetheless surprising and impactful.

## Introduction

There appears to be a growing research interest on female health practitioners who have experienced unwanted sexual harassment or abuse [1–4]. Such incidents have been reported in the literature concerning medical practitioners, but little is known about this in manual therapists. Sexual harassment is a prevalent issue faced by healthcare practitioners worldwide that results in stress and lower levels of job satisfaction [5]. It is reported that in Australia, as many as 60% of women have experienced gender-based violence or have felt unsafe in their workplace and victims are more likely to experience adverse health consequences [6]. Globally 40 to 50% of women are reported to have experience unwanted sexual advances, sexual contact or other forms of sexual harassment [7–9]. This is infrequently followed up formally [10] and rarely considered a public health concern [11] and sits at odds with societal desires to create work environments where both genders can equally survive and thrive [12].

Recent studies have termed unwelcomed sexual behaviour from patients that results in boundary violation or intimidates, humiliates or offends a practitioner as “Inappropriate Patient Sexual Behaviour” (IPSB) [13]. However, there is debate in the literature as to exactly what this means [3]. Some are obvious such as ejaculation in front of the practitioner while others are less so, such as suggestive looks or comments on dress style. Some interpret these as not being sexual harassment rather as flattery misinterpreted. Manual therapists (such as physiotherapists, chiropractors and osteopaths) are often required to engage in therapeutic interventions which involve ‘hands-on’ care. It is suggested that this may make these allied health professions especially vulnerable to IPSB [14]. There is some evidence to suggest this may be as frequent as up to 80% of respondents [4, 14, 15]. Disconcertingly, over a third of these respondents reported psychological stress effects and negative work-related consequences as a result of the IPSB [16–18]. This appears to be a significant contributor to career dissatisfaction and burnout, and may serve as an additional barrier to career advancement and retention [19].

Concerns exist as to the extent of generalizability of the published quantitative studies to all manual therapists or health professionals in Australia [14]. These studies have not explored all of the practice settings (public hospital sector, private practice, government funded care, self-funded care) [14]. In Australia, chiropractors are only employed in private practice and therefore the authors suspected that there may be a more concentrated experience of specific types of IPSB that differs from previous studies where all settings are included. There is evidence to suggest that this is the case for healthcare workers providing care outside the hospital setting, such as in patients’ homes [9]. It is thought that healthcare practitioners may, at least in part, downplay their own discomfort or distress at encounters with IPSB because of the fiduciary requirement to place patients’ best interests ahead of their own [16, 20]. The authors could find no formal understanding of how healthcare practitioners should manage IPSB systemically [3, 21]. Also, there are no specific government provided services for manual therapists experiencing harassment in Australia. Therefore, it is unsurprising that health professionals have called for guidance on setting professional boundaries, recognising violations of boundaries and information on how to deal with such incidents [14, 16, 20]. In Australia, women have reported that incidents of sexual harassment are overlooked and as a result they have little or no confidence in employers, workplace health and safety regulators capacity to act to prevent or stop IPSB occurring [11]. Such incidents have immediate negative outcomes, such as reduced productivity, increased absenteeism and reduced job satisfaction [22]. Consequently, this study aimed to address this by asking the practitioners themselves how they perceive they may be better supported moving forward [15, 16, 21].

Quantitative methods, such as questionnaires, can help identify the incidence rates and types of IPSB, and thus the effect of harassment on women’s career path [22]. Qualitative approaches afford the opportunity to explore a greater depth of understanding regarding how women in the workplace interpret and attach meaning to the incident [14, 16]. There have been several qualitative investigations exploring IPSB with healthcare workers in Australia but these have occurred within a hospital context, including nurses [23, 24], junior doctors [25], and female surgeons [26]. To the authors’ knowledge, there have been no qualitative studies focused on only female manual therapists that have captured the lived experience of IPSB in detail. The only identified study included both sexes, was mixed methods, and was published over 20 years ago [16].

Therefore, it is important to obtain a contemporary understanding of how female Australian chiropractors interpret and manage incidents of IPSB that cause them distress. As part of an Honours degree project undertaken by the second author, this study aimed to answer the following research question “What are the lived experiences of female chiropractors who have managed incidents of IPSB?”. The study had three main objectives:

1. To explore the lived experience of incidents of IPSB,
2. To characterise the types of strategies used to manage incidents of IPSB and;
3. To provide recommendations to inform professional practice.

## Method

### Methodology

The authors adopted an Interpretive Phenomenological Analysis (IPA) methodology as it was best suited to generate an understanding of the complexities, sensitivities, and subjective experience and the impact of IPSB. This methodology enables the researcher to move beyond predefined abstract categories and allow individuals to explore experiences in their own terms. IPA explores what a particular experience means to that particular individual within a particular social or cultural context [27].

The study sample was selected purposively in order to offer insight into IPSB through their ability to ‘represent’ a specified perspective. This ensures homogeneity and that patterns of similarity are captured within the group [27]. When considering the sample size, it is important to consider the richness of the individual cases. As such, there is no definitive sample size as IPA is committed to an idiographic approach. Small sample sizes of ten or less, allow the researchers to adequately capture information on meaningful points of similarity whilst maintaining differences between participants [23].

Only female participants were included in this study because experiences of IPSB are underpinned by gender inequality, gender stereotypes and discrimination; where women have become tolerant to such behaviour in our society [28, 29]. This culture of sexism and gender inequality, both inside and outside of work, has been identified as a factor responsible for reinforcing these attitudes that trivialise violence against women [6]. Women’s’ location in, and experience of, social situations are different and unequal to men's [29]. In the case of health professionals, research suggests that women tend to perceive more sexual harassment than men, with men perceiving the same incident as inappropriate rather than an actual incident [14].

### Sampling

In this study, participants were selected based on their ability to offer insight into experiences of IPSB. There were 276 female practicing chiropractic registrants in Western Australia [30]. As there was no single mailing list publicly available for this population, an electronic advertisement seeking potential participants was posted on the private Facebook pages ‘Lady Chiro’s’ (244 members) and ‘Western Australian Chiropractors and Students’ (760 members), of which the second author is a member.

Inclusion criteria was based on gender (female only), current Australian chiropractic registration status, residence (currently living in Western Australia), age (over 18 years), and has experienced IPSB in private practice. IPSB was defined in the advertisement as “an unwelcome sexual advance, unwelcome request for sexual favours or other unwelcome conduct of a sexual nature which makes a person feel offended, humiliated and/or intimidated” [13]. As an ISPB incident can be perpetrated by males or females, we did not exclude either gender as a perpetrator.

An information letter accompanying the advertisement explained the purpose of the study and stated that participants would remain anonymous. In total, nine women expressed willingness to participant in the study. Of these, one participant ceased communication and one participant was a rural practitioner and it was not feasible to conduct the interview.

The interview sample consisted of seven female chiropractors working in private practice in the Perth, Western Australia metropolitan area and were conducted from June to July of 2018. They ranged in years of experience from four to ten years (mean range of work experience 6). Participants range of working hours ranged from 9 to 28 h per week on average (mean 20.1). Direct patient consults ranged from 12 to 140 on average per week (mean 66.7) with an average patient consult duration ranging from 7.5 min to 75 min (mean consult duration 23.93 min) (see Table 1). Participants (P) were assigned a number for anonymity purposes.

**Table 1:** Primary Information

Participant (P)	Graduating year	Average hours worked per week	Average consultations per week	Average consultation duration (min)
P1	2011	24	90	15
P2	2014	28	140	7.5
P3	2010	20	45	75
P4	2014	25	55	15
P5	2013	9	35	10
P6	2008	25	90	15
P7	2014	10	12	30

The study complied with the ethical requirements of Central Queensland University, Australia (CQU ethics approval number 2018–084). Written consent was obtained from the participant, following written and verbal information about the study. Workplace or other identifiable information was not included in the transcription.

### Procedure

The semi-structured interviews were comprised of open-ended questions informed by the literature review and the professional experience of the second author. As this was a novice researcher engaged in an Honours research project, semi-structured questions were preferred. Being such a project, it was subject to time limits and funding constraints with an inability to compensate participants. This impacted on the ability to provide longer and repeated interviews; such interviews are often necessary to build rapport, gain trust and delve deeper. Also, being a novice researcher may have resulted in nervousness. Thus, the interviewer may not have been able to delve deeply into such a sensitive topic and was therefore less likely to provide the rich and detailed information necessary. To this end a practice interview and regular discussions on interview progressions were conducted. The interview was not designed to be prescriptive, rather to generate discussion and allow the participant to take the lead where appropriate. The interview aimed to explore the participant’s in-depth experience of the IPSB, the responses to these experiences, ongoing management as a result of these experiences and recommendations for support surrounding these experiences. During the interview, questions were adapted according to answers given and more interesting points were explored further. The following topic areas were included:

- Meaning of IPSB,
- Worst or most notable experience of IPSB,
- Effects and management of the incident,
- Changes in practice as a result of the incident, and
- Suggestions for education.

The face-to-face interview lasted for an average of approximately 30 min and each was conducted a location of the private practice of employment. The interviews were recorded on a digital device and then transcribed for analysis. Although it is recognised that two interviews are better especially for discussing sensitive topics, only one could be undertaken for pragmatic reasons [31]. Following transcription, all identifying information was removed and the transcript was returned to each participant for member checking to confirm the contents were a correct representation of their lived experience of IPSB. This process is important and offers novice researchers an opportunity to check the quality of their data [32] and enhances trustworthiness. All participants confirmed the contents were an accurate representation of their experience.

### Reflexivity

IPA suggests that interviews are viewed as interactions providing a snapshot of a person’s attempts to make sense of their experiences [27]. The quality of these snapshots is thought to be enhanced by establishing a rapport with the individual [33]. As such, it is important that the researcher reflects on how their own experiences will affect involvement in the interview process. Reflexivity, as it known, is central to IPA methodology and highlights personal experience as a possible undermining influence on the research. Personal disclosure and familiarity with the interview subject can facilitate a sense of empathy and encourage individuals to disclose information on a deeper level [27]. In this study, while the interviewer was a student researcher, she was also an experienced female chiropractor and therefore strongly placed to identify appropriate interview questions. Additionally, she was able to relate to the individuals on a deeper level, creating potential advantages when exploring this sensitive topic in detail. To counter any personal bias, the interviewer was debriefed after each interview with another of the other authors (a member of the Honours supervisory team and a clinical psychologist). He was also able to question whether assumptions had been made and to highlight potential areas of bias which may have been influenced by the interviewer’s background and experience.

### Analysis of interview data

The raw audio was listened to several times for inflexion and tone of comments. Additionally, the data was read several times to gain a general sense of the information as well as offering a chance to reflect on its overall meaning. Memos and notes were written in the margins of transcriptions to aid this exploration, including interpretation of the researcher’s experience of the interview itself. The transcriptions then served as a basis for the IPA analysis [27].

Data analysis followed the analytic stages proposed for IPA [34]. First, each transcript was closely read for familiarity and then emergent themes were identified, aided by annotation of significant points. Connected or related themes were then organised to create superordinate (master) themes. Emergent themes from the first transcript were then used as a guide to analyse subsequent transcripts. Once all transcripts were analysed, a table of four super-ordinate themes was constructed. The outcome being a narrative account where ‘‘the researcher’s analytic interpretation is presented in detail with verbatim extracts from participants’’ [27].

## Results

This section presents the four super-ordinate themes that emerged from the analysis; (1) familiar but inarticulable, (2) the cost of conflict, (3) I’m used to it, and (4) the element of surprise. These major themes provide insight into how female chiropractors managed IPSB and highlight the importance of better equipping female chiropractors to manage their day-to-day practice.

### Super-ordinate theme 1: familiar but inarticulable

The participants in this study demonstrated that they were intimately familiar with the issue of sexual harassment by the way they quickly and readily acknowledged the issue. However, most participants struggled to find the language to describe how the incident made them feel. Instead, they instinctively reverted to the use of familiar and commonly used emotionally laden non-descriptive language to express their response to these encounters. This is illustrated by the response of P5.

Um yeah just comments that are - would be inappropriate about - you know ahh it’s hard to explain actually.

Some participants persisted with their attempt to find words to describe the incident of IPSB. They frequently were unable to find an expression or statement that adequately described their response and they tended to use short descriptors followed by pauses before attempting another expression. This was in stark contrast to the rest of the interview in which they were articulate and without such pauses.

I felt awkward (pause), I felt irked (pause), I felt um confused. (P1)

Other participants looked to the interviewer when they were unable to express their experience and sought confirmation that although they could not find adequately find the words, the interviewer nonetheless understood what they were attempting to say and that any further descriptions were not needed. The assumption being that the interviewer understood what it meant to be a female practitioner in our current society without having to explain it further. When discussing the incident with her reception staff, P1 describes:

So, we were like ‘ooooh’. Like I suppose that’s just girls. Like that’s how we respond, right?

The inability to find adequate descriptive language persisted despite further prompting with some reverting to the use of metaphors.

Looking at both patients who were inappropriate towards me they fit a stereotype unfortunately. The body language. The - the looks I was given. The um smirk on their face. The - like that - it’s just that you could read it straight away that they were not looking at me as a professional. They were looking at me as a young female that they wanted to - yeah, a piece of meat. (P6)

### Super-ordinate theme 2: the cost of conflict

When participants were asked to describe an incident of IPSB and its impact both professionally and personally, they showed a strong tendency to dismiss or downplay the incidents. These responses were intentional and were used to actively avoid any form of confrontation with the patient. Regardless of the severity of the incident, these women expressed the view that confronting such issues would damage the professional relationship and thus the opportunity to effectively implement a therapeutic intervention with the patient.

He made this joke, ‘Oh 24 year old [name withheld] is rapeable, but 28 year old [name withheld] is not so rapeable.’ (Pause) And what did we do? We laughed about it. (Pause) How horrible is that? (P3)

Most women sought to avoid the potential of unpleasantness associated with confrontation from the patient, as well as minimizing the prospect of the possibility of a formal or informal complaint against them. As P2 explains, the repercussions of saying ‘no’ were simply not worth it.

How do you actually stand up and say no? Because you don’t want them to get defensive or try and get you [the practitioner] into trouble either. I felt like it would get super messy if I got confrontational with him.

All women in this study were willing to tolerate the incidents of IPSB to keep conflict out of the treatment room. As they reflected on this during the interview, some concluded that with the wisdom of hindsight, this conflict avoiding strategy was inappropriate. Participants described an unexpected feeling of powerlessness by employing this approach. P1 explained:

[The incident] did teach me not to be too friendly. Does that make sense? Because you do want to get along with people, and you do want them to feel comfortable when they come to the office. You want to know about their wives and their kids and things like that. So, you do - you ask questions and - you know obviously they see that you’re interested in their lives and that you are a close person in their family or community. So yeah (pause) I don’t know because I (pause) I was confused about what was happening.

P4 also recalls a significant moment of confusion when she found herself attempting to redeem the relationship by apologizing to the patient following her initiating a conversation about his inappropriate behaviour (inviting her on a date and sending gifts to her office).

So, I messaged him that night on Facebook saying ‘Thank you for the present, I’m really glad I helped you out, but it’s not appropriate to date patients.’ And then said ‘See you at your next appointment.’ Again, I didn’t want to make it awkward if he did decide to come back. [Sometime in the future] I got a phone call from him [the patient] saying to me that I made him feel uncomfortable. And that he hasn’t been seeing me because he doesn’t know how to approach me essentially. That he couldn’t come back because he felt awkward. And I was just thinking ‘What?’, I should be the one feeling awkward. It was kind of weird because I had to redeem myself and say sorry to him when really, he should have been the one saying sorry to me.

Interestingly, a few women said that they lied about personal details as an immediate means to avoid confrontation with the patient. By saying they were in a relationship, lesbian, gay, bisexual, trans, intersex or queer (LGBTIQ) the patient would no longer continue the unwanted advances. P1 explained,

What do I say? Do I just say that I’m taken (in a relationship)? Or do I just admit to [a different] sexual orientation, like, ‘No, I’m a lesbian’.

P3 said she was no longer able to dress or behave as she would like to. Rather her appearance has been directed by a desire to avoid IPSB:

I used to get my hair cut regularly and I would wear some makeup to work and I would attempt to look - you know professional – or what I thought was professional. Now that I’m [age withheld] I would say I deliberately dress like a lesbian. I actually try to make myself not attractive [to men].

Of note, several participants felt the professional associations and regulatory boards did not have procedures and guidelines in place to educate and protect its members appropriately. They felt this impacted on their ability to effectively manage the situation:

But there’s not really a guideline is there? There’s not a guideline of how to manage inappropriate comments or how to manage inappropriate physical contact. (P6)

In the absence of procedures and guidelines, most women felt that dismissal of the incident was the easiest strategy to avoid conflict. As P7 describes:

I didn’t quite know the language to use so I just kind of brushed it off.

#### Super-ordinate theme 3: i’m used to it

All participants were very tolerant of sexualised comments and behaviours from male patients, often reporting that any subsequent emotional impact was minimal. The women described these events as being so frequent across the course of their working and private adult lives that it could be described as desensitizing. P3 reported multiple incidents of IPSB and on prompting commented that these incidents were not isolated to private practice but were found in many of her previous jobs:

Some of those comments were pretty sexualised but I’ve had the experience of working in bars and there’s nothing that I haven’t been exposed to.

For other participants, desensitization was a means by which to actively avoid dealing with the inappropriate experiences. The behaviours of the men were seen to be so deeply ingrained and part of the culture they practice in, that it was impossible to change it and as such did not have a choice but to endure the IPSB. These women accepted that regardless of the emotional impact they experienced, their voices would not be heard as a result of their gender.

I’ve had other [patients] make lewd comments - you know just objectified remarks regarding women. But that’s kind of the demographic that I choose to work in anyway. So, it’s a bit like well I work in [suburb withheld], there’s a lot of miners and blue collar workers, that’s the banter. (P3)

The responses from the participants in this study suggest that there was a familiarity between the negative experiences with male patients and with males in their day-to-day experiences outside of chiropractic practice.

To some extent I think it’s because I was a female that couldn’t stand up to him - you know purely that. Whereas if I was a male he probably wouldn’t have done that in the first place. (P2)

One participant had chosen not to practice alone because she felt that this would place her at risk of a more severe incident of IPSB. This participant suggested that her gender would be the cause of any IPSB happening if alone and with a male patient.

I know that I wouldn’t put myself in that position [working in a clinic on my own]. Even someone of my size, and my age, and my personality type if a bloke decides that he’s going to do whatever he likes with you I can’t stop him. (P5)

Other participants were acutely aware of the solo nature of private practice and felt that this isolation needed to be addressed in order to better support those dealing with IPSB.

There’s not a lot of solidarity in the [chiropractic] community because a lot of us, I think, want to keep to ourselves… So, for me personally, I really don’t know actually who to turn to. (P3)

Overall, the majority of participants acknowledged that although they recognised the described incidents were inappropriate, they saw this behaviour as being out of their control and nonetheless continued their professional care.

I think there is like a - a resilience that female practitioners have to have… We accept it. Like we just have to brush it aside. (P1)

### Super-ordinate theme 4: the element of surprise

All the female chiropractors in this study, despite the number of years in clinical practice, continued to feel ill prepared to handle any situation of IPSB confidently. The issue that was most commonly spoken about was the element of unexpectedness and the surprise associated with an incident of IPSB. As P1 explains:

It [the incident] put me back a bit because you’re in this role and usually you're in control of the room.

One participant reported being surprised that the principal chiropractor and support staff had used humour to minimise her concerns. This only further reinforced the severity of gender-based inequality rather than addressing it.

I went to work the next day and I showed my boss [the text messages] and then I was showing [name withheld] - the other girl I work with - and they were both like “Oh my God, this guy,” just laughing it off. (P4)

The respondents viewed the therapeutic encounter with a clear set of expectations of the appropriate and inappropriate boundaries for the therapeutic relationship. The women offered misinterpretation of the ‘hands on’ component of chiropractic care as an explanation for crossing these boundaries. Further that the crossing of these boundaries was nearly always a surprise to the chiropractor.

You’re meant to be nice and caring to your patients etcetera but how do you really form those boundaries. All we’re really told is make sure you don’t have inappropriate relations. But if it’s not you that’s doing it and it’s the patient how do you actually stop that and where do you go? (P7)

It is therefore not surprising that the participants suggested that discussions should be occurring as part of undergraduate level training.

I think it’s something that should be broached at school [university]… We do jurisprudence and ethics and that’s where you learn all the legalities of what the AHPRA regulations are and so and so forth. However, there should also be a component in there where you have to - and it’s not just for the ladies, this is also got to be able to teach the guys as well - ensure that boundaries are met. (P5)

## Discussion

### Overarching summary

This is the first study to explore female chiropractors and lived experiences of managing incidents of IPSB. Four super-ordinate themes emerged: *familiar but inarticulable; the cost of conflict; I’m used to it; the element of surprise*. A diverse range of behaviours encountered by female chiropractors included unwanted persistent invitations for dates, unwelcome gifts, objectification, inappropriate comments and unwanted advances appearing to be the most common. While patients engaging in these behaviours represent the minority, such behaviours remain highly problematic.

#### Supra-ordinate theme 1: familiar but inarticulable

Chiropractors in this study recognised that hands-on therapy required a different set of therapeutic boundaries when compared with other non-hands-on-therapy professions. The physical contact required to conduct a physical assessment and treatment has been suggested by some as a likely contributor to the occurrence of an incident of IPSB [16]. Recently, the topic of sexual harassment has featured prominently across many media platforms. It was therefore somewhat puzzling that given its expectedness and increased social prominence that when given a forum to discuss their experiences, many lacked the appropriate language to do so. Instead, deferring to non-descriptive emotive language, metaphors and mutual understanding the topic with the interviewer as other means to describe their experience.

#### Supra-ordinate theme 2: the cost of conflict

Like harassment in other workplace settings, incidents of IPSB can have significant negative effects [5]. Emotive language used by the participants to describe the incidents included being made to feel vulnerable, embarrassed, surprised, devalued, awkward and disgusted. Immediately following the incident, and in subsequent patient visits, practitioners chose to adopt a number of different strategies to protect against a similar incident of IPSB occurring again. The most commonly implemented strategy used by the participants was to discount the incident in its entirety with avoiding conflict as the major driver for this decision. When caught off guard in a single practitioner clinic, ignoring the situation may be a viable option, however this should not be suggested as a blanket response to every situation. Ignoring the incident is more likely to lead to continuation or escalation of the behaviour, rather than putting an end to the unwanted behaviour [20, 35]. It has been suggested that when differences in authority are emphasised, such as in the doctor/patient relationship, conflict may be viewed as negative [35]. The division creates a ‘me vs. you’ environment in which conflict resolution is attempted in an accusatorial environment, rather than an environment that facilitates the accomplishment of more harmonious goals. Those who continued to see the patient did so not only to avoid this conflict within an accusatorial environment but also out of concern that the patient may lodge a formal complaint against them with the Chiropractic Board of Australia.

The participants in this study appeared to be prepared to adopt a strategy that came at a personal cost of compromise to their preferred personal boundaries. They were placing the importance of maintaining the therapeutic relationship over their personal discomfort. When an incident of IPSB occurs, the practitioner may view this as an inadequacy on their behalf to uphold the professional boundaries of the therapeutic relationship. A sense of powerlessness prevails in which the practitioner interprets the situation to then be out of her control. It was clear from the interviews that the inappropriate behaviour does not need to be persistent to diminish a practitioner’s confidence. Of course, not all participants had this reaction, with our data suggesting that some practitioners chose to dismiss isolated incidents of IPSB with minimal emotional impact.

Interestingly, a number of participants reported strategies aimed at changing their own behaviour. Self- blame has been widely documented amongst victims of sexual harassment/assault, hence it is not unusual for women to adopt strategies aimed at controlling future aversive events [36]. In our study, one woman blamed herself for the ensuing situation and took steps to redeem the relationship by apologising to the perpetrator. Other women made conscious clothing choices to look less attractive; some women changed their physical appearance by reducing makeup application and paying less attention to hairstyles when compared to their normal day-to-day routine. Believing that their physical appearance had somehow contributed to the incident occurring only further reinforces the cultural norms of gender-based discrimination in the workplace and in society [28, 37].

Other approaches to avoiding conflict included contacting the patient at a later stage to discuss the inappropriateness of the behaviour and referring the patient to a male practitioner at the same practice. Again, there appears to be a sense of professional obligation, whereby women are prepared to put their professional boundaries aside to create a non-confrontational working environment. All participants emphasised the importance of maintaining a strong rapport with the patient but reported confusion that gender had become salient in the professional working role. Samuels (2003) explains that this situation is exacerbated by a society that allows the balance of power to lie with men, regardless of the senior position of the female [38]. In conjunction with female practitioners’ socialisation as a caretaker and nurturer there presents a situation where both a females professional and gendered role may result in discomfort with assertiveness and hence the dismissal of conflict [3].

#### Supra-ordinate theme 3: i’m used to it

Sexual harassment is perceived differently with respect to the gender of the target. One consistent conclusion is that women are less tolerating of an incident when compared to men [39]. In contrast to this, our study identified that despite recognising these incidents as a violation of their personal boundaries, the female practitioners were surprisingly tolerant of the incident of IPSB. The sex role spill-over model of sexual harassment [40] postulates that working women are viewed with similar expectations of how they should behave generally. That is, male clients perceive female chiropractors as women rather than professionals, and overlook professional boundaries, treating them as a potential partner or sexual mate [21]. This could be explained as repeated exposure progressively desensitizing them until they adopted the attitude of ‘seen and heard it all before’. The message of tolerating inappropriate behaviour was also reinforced by immediate supervisors and colleagues who advised that they just ‘brush it off’. This finding is consistent with prior qualitative studies that have suggested that females are expected to adopt the ‘suck it up’ attitude [41, 42]. Such an approach reinforces the status quo and should not be tolerated. Consideration should be given to mentoring for female chiropractors, as it has been shown to help healthcare practitioners improve confidence and accountability in their efforts to address difficult practice issues [43, 44].

#### Supra-ordinate theme 4: the element of surprise

All participants appeared to presume that the adoption of the role of chiropractor after graduation would confer some type of protection from the gender stereotypes, they had become used to in their day-to-day lives. Some possible explanations may be that patients are attempting to reduce their vulnerability by minimizing the chiropractor’s authority or that it may be a manifestation of their habitual way of relating to females across all domains of life.

This assumption of respect for the role of the professional may reflect a mismatch in perceptions of the vocational status of the primary contact healthcare worker. Here the female chiropractors may over value its status while perpetrators of IPSB tend to undervalue it. Chiropractic care has been variously described, including Allied Health, as well as Complementary and Alternative Medicine (CAM) [45]. The CAM label indicating the potential for it being perceived as non-mainstream, and thus of a lower healthcare standing.

Understanding these possible explanations and revealing other reasons would require an in-depth assessment of the perpetrator; however, it is reasonable to suggest that there are a number of dynamics at play that makes not only each chiropractor’s experience with IPSB unique, but possibly the chiropractic profession as a whole.

#### Recommendations

The participants in this study were of the view that training should be implemented into the university undergraduate programs in order to be better prepared to address and respond to incidents of IPSB. This aligns with past research which found that up to 88% of Canadian and Australian physical therapists wanted undergraduate program training to this end [14, 46]. Such training results in less adverse consequences for those who did have the training [47]. Recommendations from these female chiropractors included appropriate subject matter for all chiropractic students in the Clinical or Ethics units embracing material including recognition of IPSB, creating professional boundaries, discussing the differences in gender roles as a practitioner, formal rights of the practitioner and general conflict management skills.

Not included by these women was assertiveness training, which has been found to be the most successful strategy for addressing IPSB [20, 48, 49]. This approach requires discussing the incident with the patient and subsequently setting boundaries with them. Unsuccessful strategies have been resorting to shaming, rejecting, judging or punishing the patient can result in an emotional flare up whereby the therapeutic relationship can be damaged [47]. The upside to preparing health professionals on how respond to incidents of IPSB being a reduction in adverse health outcomes [48].

Educating students and practitioners on language and managing strategies can help avoid an escalating conflict as well as for the establishment of appropriate boundaries rather than endure the IPSB. Suggested curriculum involves role-play, where the student or practitioner is given an opportunity to develop new ways of communicating in a safe environment, and where others can learn from common mistakes [20]. In addition to this formal training, it is recommended that practitioners include the use of objective documentation in their patient case files. This documentation creates a record of changes to the patient’s cognitive patterns that may occur over an observed over a period of time. Implementation of this in the day-to-day proceeding of the patient encounter can also be beneficial to address the issue with an employer or for any formal complaints that may be lodged [20]. In addition, this approach will steer practitioners away from attempting to manage such issues on a social media platform which is inappropriate for doctor-patient communication and has the potential to result in ethical charges.

On a final note, Australian national health regulatory bodies are tasked with protecting the patient from the practitioner and have created ethical guidelines to this end [50]. None exist to protect the practitioner from the patient. This concern is expressed by other healthcare disciplines such as in physiotherapy, nursing and medicine [16]. Proactive risk management is required and flags the need for future research on the topic, not only in the broader chiropractic community, but also in other health professions.

#### Strengths and limitations

In this study, IPA was considered the most appropriate methodology to address incidents of IPSB. In qualitative research, data collection relies on the skill and knowledge of the interviewer. A major limitation of the study is the potential for the novice researcher to not have been able to obtain the necessary depth of the lived experiences or have been able to establish the necessary rapport to have the participants draw out/talk freely. A skilled interviewer may be able to draw out the women’s experiences in greater detail. For example, a skilled interviewer may have been able to make better use of prompt questions and be able to draw out participant descriptions of their worst or most notable experiences of IPSB.

Other factors that may have also impacted on this study are being only able to conduct one interview, time constraints and funding limitations. Further work will need to be able to delve deeper by having repeated interviews that will allow rapport to be built which is better for sensitive subject matter. A funded study that provides compensation for the participants time could “free them up” to do this.

With respect to limitations the sample was purposively homogenous to enable exploration of IPSB across a specific sub-group. Only female participants practicing in metropolitan Western Australia were recruited and as such, the findings from this study do not represent the experiences of all chiropractors with IPSB working in private practice, irrelevant of gender. In IPA, it is important to note that the purpose of the methodology is not to generalize, but instead provide a detailed explanation of experiences from an individual perspective.

As this study was qualitative in nature and sought to explore the lived experience of IPSB, it was not possible to determine the severity and frequency of the full spectrum of incidents identified by the participants. This will likely require a quantitative methodology, such as a survey.

#### Future research

Our participants were recruited from an urban Western Australian setting. Consequently, practitioners from rural settings, other states, and other nations remain unexplored. Regional variations and cultural influences may impact on the lived experiences of IPSB. Also understanding the spectrum of IPSB will inform manual therapy educators as they seek to devise curriculum content to better prepare women for clinical practice.

It is possible that the length of consultation and treatment type may also be a factor that relates to IPSB events and warrants consideration for future investigation. This research is a starting point. Future research will need to incorporate a more detailed interview, with repeat interviews to recount as full an account as possible of their experience.

## Conclusion

This is the first study to give a forum for female chiropractors to discuss their experiences of IPSB. We describe the unique characteristics of the chiropractic doctor/patient relationship that make private practice more immune to incidents of inappropriate behaviour. Our findings also revealed practical suggestions for management strategies at both an undergraduate and professional level. We hope that this study stimulates discussion and encourages training institutions and regulatory bodies of the chiropractic profession to explore how they can better support practitioners who experience an incident of IPSB. More broadly, we hope that this study contributes in the journey toward societal change where women will no longer need to be “trained” to address unwanted behaviours. Finally, this study highlights the need for further research in the area of IPSB and practitioners in private practice.

## Data Availability

To protect participant anonymity, recordings generated during the current study are not publicly available. Deidentified transcripts are available from the corresponding author on reasonable request; however, requests will require additional Ethics approval.

## Abbreviations

CAM: Complementary and alternative medicine
CQU: Central Queensland University
IPSB: Inappropriate patient sexual behaviour
IPA: Interpretive Phenomenological Analysis
LGBTIQ: Lesbian, gay, bisexual, trans, intersex or queer
